# Introduction of Complementary Foods in a Cohort of Infants in Northeast Italy: Do Parents Comply with WHO Recommendations?

**DOI:** 10.3390/nu9010034

**Published:** 2017-01-04

**Authors:** Claudia Carletti, Paola Pani, Lorenzo Monasta, Alessandra Knowles, Adriano Cattaneo

**Affiliations:** Clinical Epidemiology and Public Health Research Unit, Institute for Maternal and Child Health IRCCS “Burlo Garofolo”, Via dell’Istria 65/1, Trieste 34137, Italy; claudiaveronica.carletti@burlo.trieste.it (C.C.); paola.pani@burlo.trieste.it (P.P.); lorenzo.monasta@burlo.trieste.it (L.M.); adriano.cattaneo@gmail.com (A.C.)

**Keywords:** complementary feeding, compliance with WHO recommendation, timing of introduction of complementary food, infant nutrition, Italy

## Abstract

Timing and type of complementary food in infancy affect nutritional status and health later in life. The objective of this paper was to assess complementary feeding practices, looking at timing, type, and compliance with World Health Organization (WHO) recommendations. Data were obtained from a birth cohort of 400 infants, enrolled in Trieste (Italy) between July 2007 and July 2008 and followed up for three years, using a “food introduction timing table”. Five WHO recommendations standards were used to assess parental compliance and associated factors. Thirty seven percent of mothers returned the completed “timing table” up until the child was three years of age. Eighty six percent of infants were already receiving complementary foods at six months. The first food type to be introduced was fresh fruit (170 days from birth, median). Overall, infants shared a very similar diet, which was different from the family diet and characterized by delayed introduction of certain food types. Five percent of parents complied with either all five or only one of the WHO recommendations, 34% with three, and 35% with four. The parents’ partial compliance with WHO recommendations is probably due to conflicting information received from different sources. This advocates for national evidence-based guidelines, supported and promoted by health professionals.

## 1. Introduction

Complementary feeding is a process that, according to World Health Organization (WHO) [1], should take place between the ages of around six months and two years, this being a crucial period for child growth, development, and health. Studies conducted in low and high-income countries show that inadequate nutrition during this period increases the risk of becoming underweight or overweight, with potentially serious life-long health effects [2,3,4,5]. Under- and over-nutrition can coexist in the same country, regardless of the country’s economic status, and tend to affect the poorest population groups [6]. The time of introduction of complementary foods, as well as their type, are crucial not only to ensure that nutritional needs are met in the short term, but also to promote good health later in life and to prevent overweight and obesity [7,8,9,10].

The WHO recommends, as a public health measure, exclusive breastfeeding up to six months of age, followed by adequate, safe and appropriate complementary foods with breastfeeding continuing up to two years and beyond [1]. This recommendation has been adopted in many countries, including Italy [11]. In conflict with the WHO recommendation, the European Society for Pediatric Gastroenterology Hepatology and Nutrition (ESPGHAN) recommends the introduction of complementary foods no earlier than 17 and no later than 26 weeks of age [12]. This commentary triggered a debate [13], and in 2011 the WHO released a statement reaffirming that exclusive breastfeeding for six months has several advantages over exclusive breastfeeding for three to four months, and should be followed by gradual introduction of solid food alongside breastfeeding [14]. WHO also recommends that, between the ages of 6 and 12 months, the infant’s diet should be progressively integrated into a healthy family diet, without restrictions on the types of food that are offered. There is no evidence to support the practice of delaying the introduction of potentially allergenic foods (e.g., tomatoes, fish, eggs), with the exception of the replacement of breast milk or formula with cow milk, which is not recommended before 12 months of age due to its low content in iron [15]. This recommendation is shared by ESPGHAN [12]. Finally, WHO warns against the use of honey in the first 12 months due to the risk of botulism, and recommends that, from six months onwards, infants should make a gradual transition to eating family foods limiting the consumption of commercial complementary foods [1,16] as these may delay the infant’s acceptance of the family’s normal diet [15].

In spite of these recommendations, data on current feeding practices suggest that the introduction of complementary foods before six months is common in many countries [17,18], including Italy [19,20], where there are no Ministry of Health guidelines on complementary feeding. The national guidelines on the protection, promotion, and support of breastfeeding simply state that “after six months, breast milk, with appropriate complementary feeding, provides an important contribution to the nutrition, health and development of the child” [11]. What “appropriate complementary feeding” means is not clearly explained.

The cohort study aimed at investigating the transition from milk to family foods, and its association with overweight, in a birth cohort of 400 children followed up for 36 months. The prevalence and duration of breastfeeding, the data on nutrient intake at six month of age and on the mothers’ socioeconomic status, have already been reported in previous papers [20,21]. The specific objective of the present research was to assess complementary feeding practices, focusing on timing, characteristics, and compliance with the WHO recommendations. Considering that there is very little national data [19,22] describing complementary feeding practices, a better understanding of parents’ approach to the introduction of complementary foods could support the development of evidence-based national guidelines on infant feeding.

## 2. Materials and Methods

Data were obtained from a cohort study conducted at the maternity hospital of Trieste (Institute for Maternal and Child Health Burlo Garofolo), Italy, between July 2007 and July 2011. A cohort of 400 mother-infant pairs was followed up for three years, using telephone interviews and self-reported diaries to investigate feeding practices and attitudes of mothers. The study was approved by the ethics committee of the Institute. The study design, methods, and sampling procedures have already been reported [21]. In brief, mother-infant pairs were enrolled at birth according to the following eligibility criteria: birth weight ≥2000 g, no congenital malformation nor severe diseases that required hospital admission, gestational age of 36 completed weeks or more, and residence in the province of Trieste. Upon enrolment, mothers were checked for eligibility and asked to give their informed consent. During the first contact, they were given a feeding diary with instructions on how to record type and quantity of foods over a 24-h period on three separate non-consecutive days at 3, 6, 9, 12, 18, 24, and 36 months of age of the infant [23,24].

At the same time, mothers were also given a “timing of introduction of food timetable” (“food timetable”) in which they were asked to record information on the characteristics, preparation and date of introduction of the first food type for each of 17 set food categories. The food timetable was handed back to the researchers when the child was 36 months old, together with the last feeding diary and the data on weight and length/height of the child recorded by the paediatricians at 1, 3, 6, 9, 12, 18, 24 and 36 months, on the occasion of periodic health checks.

The food timetable was divided into food categories: fresh fruit, vegetables (with a separate category for tomatoes because of their potential allergenicity), cereals (with a focus on gluten free pasta and bread), cow milk, milk products, meat, legumes, fish, eggs, cured meats, industrial fruit juice, tubers, honey, sugar (added to drinks or foods), sweets, creamy porridge, and nuts and seeds. For each food category, mothers were asked to record the date of the first tasting and the date from which the food type was fully incorporated in the infant’s diet, if different from the date of the first tasting. Mothers were also asked to record the method of preparation (e.g., raw or cooked, homogenized or lyophilized) and the characteristics (e.g., commercial baby food or homemade) of each food item.

WHO recommends appropriate complementary feeding starting from the age of six months, with continued breastfeeding up to two years and beyond. Appropriate complementary feeding is: timely, adequate, safe, and properly fed. On the basis of these indications we derived from the WHO recommendations five standards to assess compliance:

1. introduction at or after 6 months;
2. “minimum dietary diversity”;
3. use of homemade vs. commercial baby food;
4. introduction of cow’s milk at 12 months or more;
5. introduction of honey at 12 months or more.

Recommendation 1 describes the concept of “timeliness”, recommendations 2, 3, and 4 cover “adequateness”, meaning that food provides sufficient energy, protein, and micronutrients to meet a growing child’s nutritional needs. “Safety” is represented by recommendation 5. With regards to “propriety of feeding” the timing questionnaire did not assess either meal frequency or feeding methods.

The second recommendation is based on a slightly modified version of WHO’s “minimum dietary diversity” indicator, by which infants are defined as “compliant” if their diet incorporates four to seven different food categories by the age of 12 months. [25]. The seven food groups used by WHO to codify this indicator are: “grains, roots, and tubers”; “flesh foods (meat and fish)”; “dairy (milk) products”; “legumes and nuts”; “vitamin-A rich fruits and vegetables”; “other fruits and vegetables”; and “eggs”. In the present study, “vitamin-A rich fruits and vegetables” were not assessed as an independent group because Italy is considered to be free from vitamin A deficiency [26]. Instead, we chose to allocate to fruits and vegetables two separate categories with the aim of evaluating their independent contribution to the daily diet. Because our timing questionnaire covered a period of three years, we also extracted an index of “maximum dietary diversity” to determine how many children had introduced all seven food categories into their diet by the age of 12 months.

For the third recommendation, we analyzed the consumption of homemade vs. commercial complementary baby food defining the use of the latter as ‘high’ if it covered at least three out of five food groups (fruit, vegetables, meat, fish, cereals, and milk products) at six months of age. Almost 38% of infants fell into this category.

Differences between groups were examined with Fisher exact two-tailed test for categorical variables and with Mann-Whitney rank-sum test for continuous variables.

**Figure 1:**
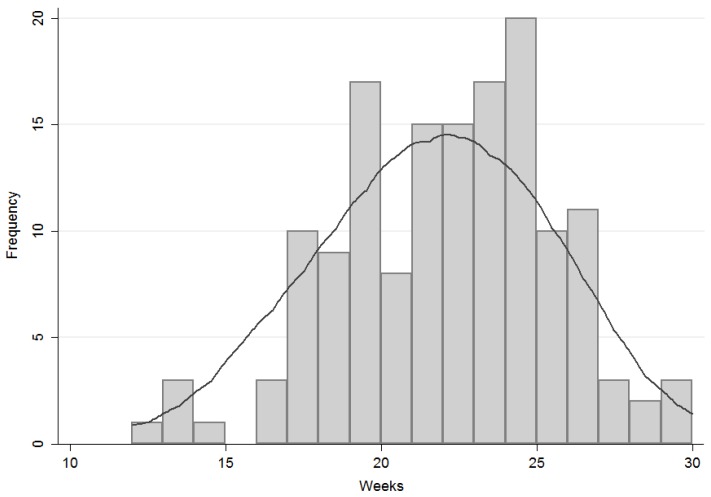
Distribution curve of the timing of first introduction of complementary foods in weeks. The probability density function was estimated with a non-parametric Kernel density estimation (KDE).

In order to perform the logistic regression analyses described below, the sample was divided into two groups based on the timing of the first introduction of complementary food. The cut-off was set at 21 weeks, which was the median of the distribution curve (Figure 1). The 21 weeks value had the additional advantage of falling in the middle of the ESPGHAN recommended window (17 to 26 weeks). This timing distribution was used as dependent variable.

The independent variables reflected the objective of the study to assess compliance with WHO recommendations both as stand-alone variables and as overall compliance with any three out of five. The relation between these independent variables and timing was analyzed using stratified and simultaneous univariate and multivariate logistic regression analyses. The variables included in the simultaneous models are presented in Supplementary Materials Tables S1–S6.

The stratified analysis was carried out considering each of the following variables: mothers’ characteristics, including age (≥34 years), body mass index (BMI) before pregnancy (≥25), place of birth (Italy vs. other), level of education (degree or higher vs. lower), occupation, whether she was breastfeeding or had breastfed, if she had allergies or a family history of allergies, if she was or had been a smoker, and type of professional figure (paediatrician or midwife) who provided counselling on complementary feeding.

The characteristics of the mothers were selected based on literature data. The information on which professional figures were providing counselling on complementary feeding was based on the hypothesis, formulated by the authors but also supported by literature [27,28], that this may influence mothers’ behavior.

All the analyses were carried out using Stata IC 14.1 (StataCorp LP, College Station, TX, USA).

## 3. Results

### 3.1. Population Characteristics

Four hundred mother-infant pairs were enrolled in the study, but only 148 (37%) completed and returned the food timetable when the child reached 36 months of age. The participation rate declined during the study: after three months 34% (135/400) of mothers had withdrawn from the study. This percentage subsequently followed a fluctuating pattern, rising to 59% (235/400) at 6 months and then dropping to 43% (173/400) and 41% (165/400) at 9 and 12 months, respectively. By the time of the 24-month interview, the study cohort had reduced by 67% (132/400). The characteristics of the mothers and infants of the sub-cohort that completed the timing questionnaire are described in Table 1. No statistically significant differences were observed between the cohort (400) and the sub-cohort (148), as already reported [16,17]. In brief, 89% of mothers (mean age 33.7 years; Standard Deviation 4.4) had a medium-to-high level of education, 95% declared they were employed before birth and 80% were in employment at six months after birth. Most infants (90%) were born between 38 and 42 weeks of gestation and 82% by vaginal delivery. The majority (91%) weighed between 2500 g and 4199 g at birth, and 79% were between 46 and 52 cm long. The infants’ mean BMI was calculated at three and six months: 70% and 75% of infants had a BMI that fell within the normal range (between the 15th and the 85th WHO percentile), 15% and 3% were below the 15th percentile, while 15% and 21% were over the 85th percentile, respectively.

### 3.2. Timing of Complementary Feeding

At six months of age, 73% of infants were still receiving breast milk but, in contrast with WHO recommendations, only 7% were exclusively breastfed; 40% received formula and breast milk, 27% only formula and 26% breast milk and complementary food. One infant was given food other than milk before the age of three months, 7% at three months, 32% at four, 47% at five months. Only 14% of infants were given their first complementary food at six months or more

**Figure 2:**
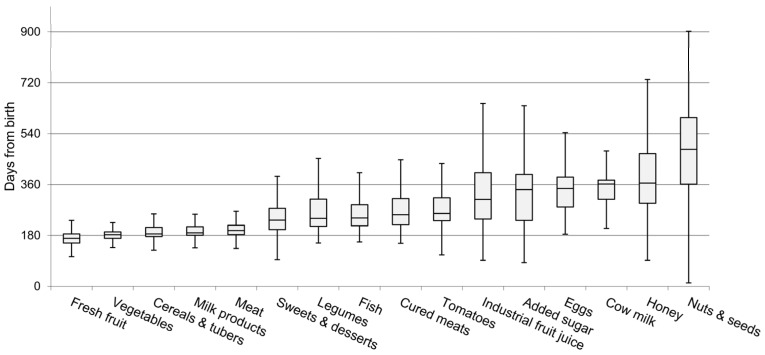
Timing of introduction of the single food groups in days from birth. The ends of the whiskers are set at 1.5*IQR above the third quartile (Q3) and 1.5*IQR below the first quartile (Q1). If the Minimum or Maximum values are outside this range, then they are shown as outliers. IQR = Inter Quartile Range.

The median age of introduction of solid foods was 5.2 months (min 2.8; max 7.2). The interval between the first tasting and the complete introduction of the different food categories ranged from 6 to 32 days. The range was narrower for food groups such as dairy products, vegetables, and cereals (6, 6, and 7 days, respectively) and wider for honey, sugar, and cow milk (32, 29, and 22 days, respectively). The timetable of the complete introduction of each food category is reported in Figure 2.

In the figure, the cereals/tubers categories and the sweets/desserts categories, which include sweets and creamy porridge, have been merged for representational purposes. The first food group to be introduced was fresh fruit (at a median age of 170 days); followed by vegetables (182 days); cereals, including bread, pasta with gluten, and rice (193 days); milk products (189 days); and meat (197 days). The last food groups to be introduced were cow milk (362 days), honey (365 days), and nuts and seeds (484 days). In 80% of the cases, the first type of food to be offered was the apple, followed by vegetable soup (41%), and baby cereals (41%). The use of gluten-free products (bread, pasta, and desserts) was low (17% of infants) and the timing of their introduction was similar to that of the products with gluten. The introduction of pasta with gluten occurred at a median of 234 days, as opposed to 224 days for gluten-free pasta. The most frequently used commercial baby food types were: milk products (67%), fish (62%), sweets and desserts (61%), cured meat (52%), cereals (52%), meat (49%), fruit (27%), and vegetables (3%).

### 3.3. Compliance with WHO Recommendations

Table 2 shows the percentage of mothers who complied with the WHO recommendations on complementary feeding. The recommendations to which most mothers adhered were the minimum diversity diet (96%) and the introduction of honey after 12 months of age (80%), while 62% of the mothers complied with the recommendation to limit the use of commercial baby foods. However, the analysis of the “maximum diversity diet” indicated that, by the age 12 months, only 45% of children had introduced all seven food groups and, in particular, eggs and legumes had been introduced by 53% and 74% of infants, respectively.

**Figure 3:**
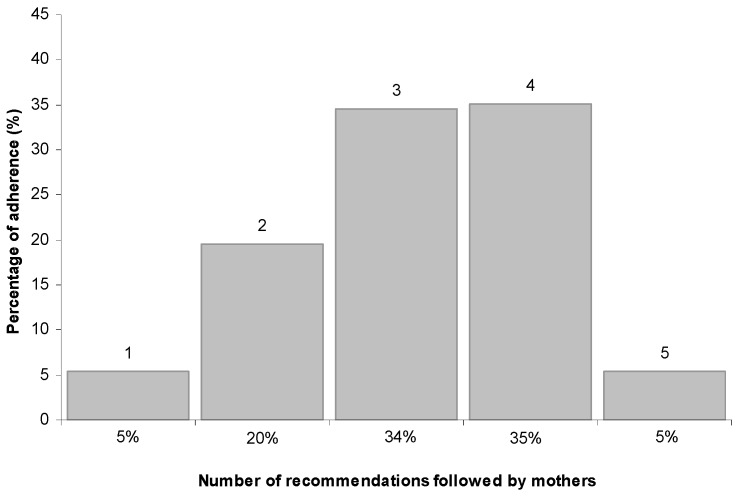
Number of recommendations (0, 1, 2, 3, 4 and 5) followed by mothers and percentage of compliance.

As shown in Figure 3, eight mothers (5%) complied with all five recommendations and eight (5%) complied with only one, while 34% complied with at least three recommendations. Within this subgroup, the least frequently adhered to recommendation was the introduction of complementary feeding after the age of six months (19%).

### 3.4. Regression Analyses

The stratified analyses showed that only few of the outcomes considered were significantly associated with selected mothers’ characteristics. The introduction of cow milk after 12 months was significantly associated with the introduction of the first foods at 21 weeks of age or more if mothers were not breastfeeding their infants at six months (66%, *p* = 0.03), did not have a family history of allergy (80%, *p* < 0.01), had a BMI (kg/m^2^) of less than 25 (72%, *p* = 0.02), and were younger than 35 years old (76%, *p* = 0.02). The test of homogeneity showed significant differences only between mothers with or without a history of allergy (*p* = 0.03).

Compliance with at least three out of five recommendations was significantly associated with the introduction of the first food at 21 weeks of age or more, if mothers where not breastfeeding their infants at six months (63%, *p* = 0.05), did not have a family history of allergy (74%, *p* = 0.01), had a BMI (kg/m^2^) of less than 25 (71%, *p* = 0.02), and if they had received information on infant feeding from a midwife (68%, *p* = 0.03).

The multivariate logistic regression analyses showed no significant associations between the outcomes and maternal characteristics, probably due to the small size of the sample. The results are reported in the Supplementary Materials section (Tables S2–S6).

## 4. Discussion

As already observed in other studies [20,22,29], the timing of the introduction of complementary foods tends to follow a standard pattern: the infant’s diet includes only few categories of food and differs substantially from that of the family, with commercial baby foods taking up a considerable share. Our study shows that the feeding practices of this cohort follow the same pattern and fall short of the WHO recommendations on breast and complementary feeding. Seventy five percent of mothers adhered to three or more out of five WHO recommendations on adequate complementary feeding, but only 5% complied with all five. The lowest rate of compliance related to the recommendation to introduce complementary foods at six months or more, with 86% of infants receiving complementary foods before the age of six months. This percentage is higher than the one reported in a recent study in the Netherlands [14], but close to the data reported in a nutrition survey from the UK [30] and in a WHO multicenter study of comparable sample size conducted in different countries [31]. However, comparisons with other studies are difficult because most of them adopt a cut-off of 17 weeks of age for early introduction, as recommended by ESPGHAN [12].

Despite the evidence that, in terms of prevention of allergies, there is no benefit in delaying the introduction of potentially allergenic foods [15] and that, in fact, early contact with food can induce tolerance and desensitization [32], the mothers in our cohort deferred the introduction of tomatoes, eggs, fish, and nuts and seeds, to between 8 (fish) and 16 months of age (nuts and seeds), as shown in Figure 2. This may have contributed to the lack of “maximum dietary diversity” by 12 months observed in more than half of the infants in our cohort. Indeed, as noted in a recent national survey [33,34], in a developed country such as Italy, without nutritional deficiencies and with high availability of nutrients, the “maximum dietary diversity” index could, and should, be higher.

It has been shown that the process of food learning starts very early and that there is a period in which new foods are relatively easily accepted [35,36]. Because food preferences developed at an early age may have long-lasting influence [37], it would be desirable to expose infants to as many tastes as possible in the first year of life. In her study [38], Cashdan showed that infants introduced to solids unusually late maintain a reduced diet and food range throughout childhood, perhaps as a result of the contraction of the sensitive period.

The partial compliance with WHO recommendations observed in our cohort, has already been reported by others [22,29,39]. Considering that 98% of the mothers in our study declared they received information on complementary feeding from their paediatrician and that in 62% of the cases, this advice was followed, the role of health professionals seems to be crucial, despite the lack of a statistically significant association in our regression analyses.

One of the possible reasons for the lack of correct and consistent information on the timing and type of complementary feeding is that in Italy there are no specific national Ministry of Health guidelines incorporating the WHO recommendations. These are therefore unevenly acquired by health professionals and poorly transferred to parents.

To our knowledge, this is the first study to explore in detail timing of complementary feeding and compliance with WHO recommendations, and associated factors, in Italy. The study does, however, have a number of limitations. The main problem is the loss to follow up of enrolled mother-infant pairs between birth and 36 months of age (37%), which greatly reduced the availability of complete data and thus the power to detect significant associations by multivariate analyses. The second major limitation is the complexity of the food timetable which required regular updating over a long period of time with detailed information regarding newly introduced food types. Although this tool is designed to provide high quality data, in our case this was at the expense of the sample size, making it difficult to compare our results with those of other national surveys.

## 5. Conclusions

Our paper describes the partial compliance with WHO recommendations on complementary feeding, in northeast Italy. Most of the infants in our cohort were started on complementary foods before the age of six months. They shared a very similar diet, which was very different from the family diet and characterized by the delayed introduction of certain food types. Further research will be needed to clarify the association between compliance with recommendations and socio-economic variables, in order to identify vulnerable groups in the population, including migrant families.

Taken together, our results suggest that Italy would greatly benefit from national evidence-based guidelines on infant feeding to support the development of solid public health strategies. These guidelines should be free from commercial interests, consistently adhered to, and promoted by all health professionals, especially in the light of the considerable influence their advice seems to have on parental practices.

## Figures and Tables

**Table 1:** Characteristics of mothers at enrolment and children at birth.

	Cohort	Sub-Cohort	
**Mothers**	***n* (%)**	***n* (%)**	*p* *
Age (years)	(*n* = 399)	(*n* = 148)	0.713
≤29	90 (23)	29 (20)	
30–34	160 (40)	59 (40)	
≥35	149 (37)	60 (40)	
Born in Italy	(*n* = 400)	(*n* = 148)	0.451
Yes	351 (88)	134 (91)	
No	49 (12)	14 (9)	
Education	(*n* = 348)	(*n* = 147)	0.158
≤Secondary school	59 (17)	16 (11)	
Completed high school or equivalent	154 (44)	64 (43)	
Bachelor degree or higher	135 (39)	67 (46)	
Employment before birth	(*n* = 323)	(*n* = 136)	1.000
Yes	306 (95)	129 (95)	
No	17 (5)	7 (5)	
Employment at 6 months after birth	(*n* = 218)	(*n* = 147)	0.790
Yes	176 (81)	117 (80)	
No	42 (19)	30 (20)	
Allergy of mother or of other family member	(*n* = 263)	(*n* = 148)	0.812
Yes	64 (24)	38 (26)	
No	199 (76)	110 (74)	
**Children**	***n* (%)**	***n* (%)**	***p***
Gestational age (weeks)	(*n* = 339)	(*n* = 147)	0.730
36–37	29 (9)	14 (9)	
38–42	310 (91)	133 (91)	
Infant gender	(*n* = 345)	(*n* = 148)	0.142
male	173 (50)	85 (57)	
female	172 (50)	63 (43)	
Birth weight (g)	(*n* = 344)	(*n* = 148)	0.552
<2500	3 (1)	2 (1)	
2500–4199	324 (94)	136 (92)	
≥4200	17 (5)	10 (7)	
Birth length (cm)	(*n* = 342)	(*n* = 148)	0.563
<46	5 (1)	2 (1)	
46–52.9	283 (83)	117 (79)	
≥53	54 (16)	29 (20)	

* *p*-Value is calculated using a two-tailed Fisher Exact test.

**Table 2:** Percentage of mothers who followed the WHO recommendations (*n* = 148).

Items Included in the Score	(*n*)
Introduction of solid foods ≥6 months	14% (21)
Reduced use of commercial baby foods	62% (92)
Introduction of cow’s milk ≥12 months	63% (94)
Introduction of honey ≥12 months	80% (118)
Minimum dietary diversity	96% (142)

## Supplementary Materials

The following are available online at http://www.mdpi.com/2072-6643/9/1/34/s1. Table S1: Association between introduction of first foods after 21 weeks and compliance with WHO recommendations both as stand-alone variables and as overall compliance with any three out of five and selected variables by bivariate logistic regression analysis (*n* = 148), Table S2: Association between introduction of first foods after 21 weeks and introduction of honey after 12 months adjusted by selected variables by multivariate logistic regression analysis (*n* = 130), Table S3: Association between introduction of first foods after 21 weeks and introduction of cow milk after 12 months adjusted by selected variables by multivariate logistic regression analysis (*n* = 130), Table S4: Association between introduction of first foods after 21 weeks and less consumption of baby food adjusted by selected variables by multivariate logistic regression analysis (*n* = 130), Table S5: Association between introduction of first foods after 21 weeks and minimum dietary diversity recommendation adjusted by selected variables by multivariate logistic regression analysis (*n* = 130), Table S6: Association between introduction of first foods after 21 weeks and compliance with three out of five WHO recommendations adjusted by selected variables by multivariate logistic regression analysis (*n* = 130).
