# Supplementary material for: Introduction of Complementary Foods in a Cohort of Infants in Northeast Italy: Do Parents Comply with WHO Recommendations?
Source: Nutrients. 2017 Jan 4;9(1):34. doi: 10.3390/nu9010034 (PMC5295078; doi:10.3390/nu9010034)
Supplement: Supplementary file 1 [file nutrients-09-00034-s001.docx]

Supplementary Materials: Introduction of Complementary Foods in a Cohort of Infants in Northeast Italy: Do Parents Comply with WHO Recommendations?

Claudia Carletti, Paola Pani, Lorenzo Monasta, Alessandra Knowles and Adriano Cattaneo

**Table S1.** Association between introduction of first foods after 21 weeks and compliance with WHO recommendations both as stand-alone variables and as overall compliance with any three out of five and selected variables by bivariate logistic regression analysis (*n* = 148).

| **Timing of Introduction of First Food >21 Weeks** | **Odds Ratio** | **95% CI** | ***p*** |
| --- | --- | --- | --- |
| Honey after 12 months | 0.80 | 0.35–1.81 | 0.592 |
| Cow milk after 12 months | 0.49 | 0.24–0.98 | 0.045 |
| Less consumption of baby foods | 1.30 | 0.64–2.61 | 0.468 |
| Minimum dietary diversity | 2.17 | 1.09–4.30 | 0.027 |
| Compliance with three out of five WHO recommendations | 0.60 | 0.30–1.18 | 0.139 |
| Breastfeeding at six months | 0.53 | 0.25–1.13 | 0.102 |
| Allergies or a family history of allergies | 1.36 | 0.69–2.68 | 0.381 |
| Mother smoker | 1.45 | 0.51–4.16 | 0.485 |
| Overweight/Obesity before pregnancy | 1.05 | 0.45–2.46 | 0.911 |
| Schooling (degree or higher vs. lower) | 0.61 | 0.30–1.20 | 0.153 |
| Age ≥34 years | 0.58 | 0.29–1.14 | 0.116 |
| Information given by pediatrician | 1.12 | 0.10–12.74 | 0.923 |
| Information given by midwife | 1.32 | 0.62–2.78 | 0.467 |
| Place of birth (Italy vs. other) | 0.72 | 0.23–2.20 | 0.565 |
| Occupation (yes vs. no) | 1.35 | 0.57–3.22 | 0.491 |

WHO: World Health Organization; CI: confidence interval.

**Table S2.** Association between introduction of first foods after 21 weeks and introduction of honey after 12 months adjusted by selected variables by multivariate logistic regression analysis (*n* = 130).

| **Timing of Introduction of First Food >21 Weeks** | **Odds Ratio** | **95% CI** | ***p*** |
| --- | --- | --- | --- |
| Honey after 12 months | 0.82 | 0.30–2.22 | 0.695 |
| Breastfeeding at six months | 0.45 | 0.12–1.13 | 0.090 |
| Allergies or a family history of allergies | 1.24 | 0.55–2.82 | 0.600 |
| Mother smoker | 1.62 | 0.44–5.95 | 0.468 |
| Overweight/Obesity before pregnancy | 1.34 | 0.49–3.72 | 0.569 |
| Schooling (degree or higher vs. lower) | 0.59 | 0.25–1.37 | 0.224 |
| Age ≥34 years | 0.79 | 0.34–1.78 | 0.570 |
| Information given by pediatrician | 1.73 | 0.13–22.62 | 0.675 |
| Information given by midwife | 1.44 | 0.62–3.35 | 0.392 |
| Place of birth (Italy vs. other) | 0.38 | 0.09–1.53 | 0.174 |
| Occupation (yes vs. no) | 1.41 | 0.49–4.13 | 0.520 |

**Table S3.** Association between introduction of first foods after 21 weeks and introduction of cow milk after 12 months adjusted by selected variables by multivariate logistic regression analysis (*n* = 130).

| **Timing of Introduction of First Food >21 Weeks** | **Odds Ratio** | **95% CI** | ***p*** |
| --- | --- | --- | --- |
| Cow milk after 12 months | 0.46 | 0.21–1.04 | 0.063 |
| Breastfeeding at six months | 0.50 | 0.20–1.24 | 0.135 |
| Allergies or a family history of allergies | 1.36 | 0.60–3.07 | 0.456 |
| Mother smoker | 1.61 | 0.42–6.17 | 0.484 |
| Overweight/Obesity before pregnancy | 1.52 | 0.54–4.32 | 0.427 |
| Schooling (degree or higher vs. lower) | 0.57 | 0.24–1.34 | 0.196 |
| Age ≥34 years | 0.81 | 0.35–1.85 | 0.612 |
| Information given by pediatrician | 2.10 | 0.15–29.86 | 0.584 |
| Information given by midwife | 1.69 | 0.71–4.05 | 0.236 |
| Place of birth (Italy vs. other) | 0.33 | 0.08–1.37 | 0.128 |
| Occupation (yes vs. no) | 1.44 | 0.49–4.19 | 0.504 |

**Table S4.** Association between introduction of first foods after 21 weeks and less consumption of baby food adjusted by selected variables by multivariate logistic regression analysis (*n* = 130).

| **Timing of Introduction of First Food >21 Weeks** | **Odds Ratio** | **95% CI** | ***p*** |
| --- | --- | --- | --- |
| Less consumption of baby foods | 1.19 | 0.53–2.71 | 0.670 |
| Breastfeeding at six months | 0.46 | 0.19–1.13 | 0.092 |
| Allergies or a family history of allergies | 1.26 | 0.56–2.82 | 0.571 |
| Mother smoker | 1.55 | 0.41–5.82 | 0.513 |
| Overweight/Obesity before pregnancy | 1.38 | 0.49–3.88 | 0.541 |
| Schooling (degree or higher vs. lower) | 0.60 | 0.26–1.39 | 0.235 |
| Age ≥34 years | 0.79 | 0.35–1.78 | 0.565 |
| Information given by pediatrician | 1.60 | 0.12–20.85 | 0.718 |
| Information given by midwife | 1.44 | 0.62–3.34 | 0.393 |
| Place of birth (Italy vs. other) | 0.38 | 0.09–1.51 | 0.169 |
| Occupation | 1.37 | 0.47–3.98 | 0.567 |

**Table S5.** Association between introduction of first foods after 21 weeks and minimum dietary diversity recommendation adjusted by selected variables by multivariate logistic regression analysis (*n* = 130).

| **Timing of Introduction of First Food >21 Weeks** | **Odds Ratio** | **95% CI** | ***p*** |
| --- | --- | --- | --- |
| Minimum dietary diversity | 2.09 | 0.94–4.62 | 0.068 |
| Breastfeeding at 6 months | 0.43 | 0.17–1.07 | 0.070 |
| Allergies or a family history of allergies | 1.17 | 0.52–2.64 | 0.697 |
| Mother smoker | 1.72 | 0.46–6.44 | 0.417 |
| Overweight/Obesity before pregnancy | 1.20 | 0.43–3.33 | 0.722 |
| Schooling (degree or higher vs. lower) | 0.61 | 0.26–1.43 | 0.256 |
| Age ≥34 years | 0.76 | 0.33–1.74 | 0.517 |
| Information given by pediatrician | 2.19 | 0.15–31.55 | 0.563 |
| Information given by midwife | 1.41 | 0.60–3.31 | 0.423 |
| Place of birth (Italy vs. other) | 0.39 | 0.10–1.60 | 0.192 |
| Occupation | 1.38 | 0.46–4.12 | 0.558 |

**Table S6.** Association between introduction of first foods after 21 weeks and compliance with three out of five WHO recommendations adjusted by selected variables by multivariate logistic regression analysis (*n* = 130).

| **Timing of Introduction of First Food >21 Weeks** | **Odds Ratio** | **95% CI** | ***p*** |
| --- | --- | --- | --- |
| Compliance with three out of five WHO recommendations | 0.52 | 0.24–1.16 | 0.111 |
| Breastfeeding at 6 months | 0.48 | 0.19–1.20 | 0.116 |
| Allergies or a family history of allergies | 1.33 | 0.59–2.99 | 0.484 |
| Mother smoker | 1.78 | 0.47–6.75 | 0.395 |
| Overweight/Obesity before pregnancy | 1.29 | 0.46–3.63 | 0.625 |
| Schooling (degree or higher vs. lower) | 0.56 | 0.24–1.32 | 0.186 |
| Age ≥34 years | 0.78 | 0.34–1.78 | 0.564 |
| Information given by pediatrician | 1.85 | 0.14–23.83 | 0.637 |
| Information given by midwife | 1.53 | 0.65–3.59 | 0.331 |
| Place of birth (Italy vs. other) | 0.37 | 0.09–1.49 | 0.161 |
| Occupation | 1.60 | 0.54–4.72 | 0.391 |
